# A Rare Pediatric Encounter: Transverse Myelitis With Subacute Bilateral Lower Extremity Weakness

**DOI:** 10.7759/cureus.46041

**Published:** 2023-09-26

**Authors:** Aleena Arif, Quratulain Fatima Masood, Nadia M Chowdhury, Maryam Affaf, Gavitha N Thabrew Wijeratne

**Affiliations:** 1 Internal Medicine, Allama Iqbal Medical College, Lahore, PAK; 2 Surgery, National University of Science and Technology, Rawalpindi, PAK; 3 Medicine, Ross University School of Medicine, Frederick, USA; 4 Internal Medicine, Women’s Medical and Dental College, Abbotabad, PAK; 5 Medicine, Ross University School of Medicine, New York, USA

**Keywords:** thoracic spinal cord, high-dose intravenous corticosteroids, diagnostic evaluations, pediatric, transverse myelitis

## Abstract

This case report provides a comprehensive overview of a rare instance of transverse myelitis (TM) in an 11-year-old male who presented with subacute bilateral lower extremity weakness, sensory loss, and bowel/bladder dysfunction. Diagnostic evaluations, including MRI and cerebrospinal fluid (CSF) analysis, confirmed TM. Management with high-dose intravenous corticosteroids and rehabilitative therapies led to symptom stabilization and modest recovery, although some permanent deficits are anticipated. The report highlights the importance of prompt diagnosis and intervention in pediatric patients with acute neurologic symptoms localized to the spinal cord. It contributes to the existing literature by detailing the clinical and diagnostic features of pediatric TM, aiding in early recognition and management to minimize permanent disability. Future research should focus on understanding the underlying pathophysiology with a view to developing targeted therapies.

## Introduction

Transverse myelitis (TM) is an acquired, localized inflammatory disorder that targets the spinal cord. It leads to a range of neurological deficits, including paralysis, sensory alterations, and issues with bladder and bowel control [1]. The condition often manifests with a rapid onset of symptoms such as discomfort, numbness, tingling, limb weakness, and autonomic dysfunction [2]. The severity of these symptoms can vary widely among individuals. TM is a rare inflammatory demyelinating condition that predominantly affects adults, with children being less commonly impacted [3].

The objective of this case report is to provide comprehensive documentation of a rare pediatric case of TM. The report aims to offer broader context and insights into the condition, by detailing the course of the disease, its management, and patient outcomes.

## Case presentation

An 11-year-old male patient was brought to the pediatric emergency department with a three-day history of lower limb weakness that had been sudden in onset, progressive, non-exertional, and symmetrical. The symptoms were accompanied by fecal and urinary retention. The patient had experienced a common cold 15 days prior but had no history of diarrhea, vomiting, trauma, back pain, fever, weight loss, vision loss, intramuscular injections, nasal regurgitation, voice changes, or respiratory distress. He had never been hospitalized or undergone surgery. The child had been born full-term via spontaneous vaginal delivery with no history of delayed cry, cyanosis, birth asphyxia, or NICU admission. Vaccinations were up to date according to the Expanded Programme on Immunization (EPI) schedule, and a BCG scar was present. There was no family history of similar illness, tuberculosis, or poliomyelitis.

Upon examination, the patient's vitals were stable with a pulse of 84/minute, blood pressure of 100/60 mmHg, temperature of 98 °F, and respiratory rate of 26/minute. Neurological examination revealed a Glasgow Coma Scale (GCS) score of 15/15, and all cranial nerves were intact. Motor examination of the upper limbs showed normal tone, power, and reflexes. However, the lower limbs exhibited decreased tone, power of 1/5, and absent reflexes. Sensory examination revealed absent sensations of fine touch, pain, temperature, and vibration in the lower limbs, with a sensory level evident at T10. A gastrointestinal examination showed a non-distended and soft abdomen with no visceromegaly, and the bladder was palpable.

**Figure 1:**
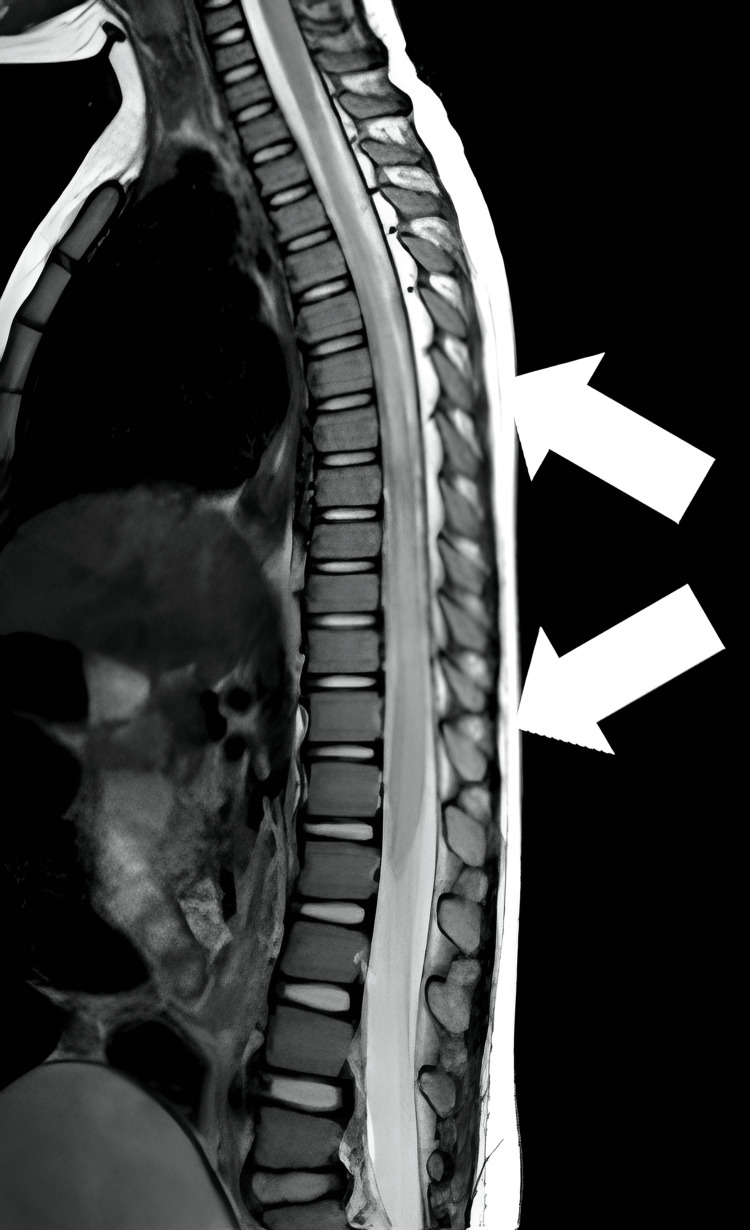
MRI of the whole spinal cord without IV contrast The image showed a long segment of abnormal T2W and FLAIR hyperintense signals in the thoracic cord from T5 to T11 MRI: magnetic resonance imaging; FLAIR: Fluid-attenuated Inversion-Recovery

Based on these findings, the differentials considered were TM, Guillain-Barré syndrome, and hypokalemic paralysis. Investigations were started with complete blood count (CBC), liver function tests (LFTs), and renal function tests (RFTs). The details are provided in Table 1. Further investigations included an MRI of the whole spinal cord without IV contrast, which showed a long segment of abnormal T2W and FLAIR hyperintense signals in the thoracic cord from T5 to T11 (Figure 1). MRI of the brain and orbit was normal. The patient tested negative for anti-aquaporin 4 antibodies. Cerebrospinal fluid (CSF) testing showed a clear appearance with 12 cells/mm³ of WBCs (100% lymphocytes), glucose level of 70 mg/dl, and protein level of 45 mg/dl. The findings are presented in Table 2. Electrophysical studies were within normal limits.

**Table 1:** Blood workup of the patient INR: international normalized ratio; aPTT: activated partial thromboplastin time; WBC count: white blood cell count; RBC: red blood cells; HCT: hematocrit; MCV: mean corpuscular volume; MCH: mean corpuscular hemoglobin; MCHC: mean corpuscular hemoglobin concentration; ALT: alanine transaminase; AST: aspartate aminotransferase; ALP: alkaline phosphatase; ESR: erythrocyte sedimentation rate

Coagulation profile		
		Reference range
Prothrombin time-control	11	10-14 seconds
Prothrombin time-patient	12	Up to 13 seconds
INR	1.1	0.9-1.3
Control time	26	25-35 seconds
aPTT	28	Up to 31 seconds
Hemogram		
WBC count	5 x 10^9^	4-11 x 10^9^/L
Total RBC	4.1	3.8-5.2 x 10^12^/L
Hemoglobin	13.3	13-18 (g/dL)
HCT	37	35-46%
MCV	80	77-95 fl
MCH	30	26-32 pg
MCHC	34	32-36 g/dL
Platelets	366	150-400 x 10^9^/L
Neutrophils	47	40-80%
Lymphocytes	31	20-40%
Monocytes	5	2-10%
Eosinophils	3	1-6%
Renal function tests		
Urea	27	10-50 mg/dl
Serum creatinine	0.6	0.5-0.9 mg/dl
Liver function tests		
Bilirubin total	0.7	0.3-1.2 mg/dl
Total protein	6.5	5.7-8.2 g/dl
Albumin	3.9	3.2-4.8 g/dl
ALT	35	Up to 40 U/L
AST	30	Up to 40 U/L
ALP	79	40-120 U/L
Serum electrolytes		
Sodium	140	135-145 mmol/L
Potassium	4.5	3.5-5 mmol/L
Chloride	105	98-107 mmol/L
Calcium	9.2	8.5-10.5 mg/dl

**Table 2:** Cerebrospinal fluid analysis findings

Parameter	Value	Reference range	Interpretation
Appearance	Clear	Clear	Normal
WBC count, cells/mm³	12	0-5	Elevated
Lymphocyte percentage	100%	Varies	Predominantly lymphocytes
Glucose level, mg/dl	70	40-70	Within normal limits
Protein level, mg/dl	45	15-45	Within normal limits

In this case, the MRI findings played a critical role in confirming the diagnosis of long-segment TM. The T2-weighted (T2W) images were particularly illuminating, revealing a long segment of abnormal hyperintense signals in the thoracic spinal cord from T5 to T11. T2W imaging is highly sensitive for detecting changes in tissue characteristics, such as inflammation or demyelination, as it makes fluid appear bright. The abnormal T2W signals in the thoracic cord were indicative of a pathological change, likely due to inflammation. These T2W findings were instrumental in corroborating the clinical symptoms of lower limb weakness and urinary and fecal retention and provided valuable insights into the extent and specific location of the spinal cord involvement. This information was crucial for guiding the targeted treatment approach, ultimately contributing to the patient's favorable prognosis.

The patient was managed with intravenous methylprednisolone at a dose of 30 mg/kg/day for five days, along with supportive treatments including analgesics (a combination of acetaminophen and gabapentin), symptomatic management, and bladder and bowel care. Bladder and bowel care were essential components of the patient's management, involving intermittent catheterization for urinary retention and a bowel regimen to address fecal retention. These interventions aimed to prevent complications such as urinary tract infections and constipation.

Rehabilitation included physiotherapy, occupational therapy, and psychotherapy. The patient's symptoms improved significantly with this management, and he is currently on a course of medications and living a healthy life. The prognosis was favorable. The patient was counseled about the nature of the ailment and the importance of regular follow-up was emphasized.

## Discussion

TM is an inflammatory disorder affecting the spinal cord, leading to motor, sensory, and autonomic dysfunction below the level of the affected cord segment [4]. Our patient exhibited a loss of tone and motor function in the lower limbs, which aligns with typical presentations of TM. The estimated incidence of TM in pediatric populations ranges from one to eight per million children per year. Interestingly, the age of onset in children follows a bimodal distribution, peaking between one and eight years and 14 and 16 years [3]. Our patient, being 11 years old, falls within the early childhood peak, further corroborating the typical age-related incidence of TM.

Weakness is the most prevalent symptom of TM, occurring in 77-89% of TM patients, and it predominantly affects the legs. Our patient's clinical presentation was consistent with this, as he experienced significant lower limb weakness. Other classic symptoms include sensory loss (48-68%), bladder or bowel dysfunction (28-59%), and pain (29%). The extent of the spinal cord involvement and the severity of inflammation directly influence the degree of these deficits [4]. Our patient also experienced fecal and urinary retention, which is another hallmark of TM.

The absence of anti-aquaporin 4 antibodies in our patient's test results holds diagnostic and prognostic implications. Specifically, this absence effectively rules out neuromyelitis optica spectrum disorders (NMOSD), a group of conditions that can mimic the symptoms of TM but often require a different treatment approach and have a less favorable prognosis. The lack of these antibodies suggests that the transverse myelitis in this case is likely idiopathic, meaning it is not associated with any other known disease or condition. This information not only narrows down the differential diagnosis but also guides the treatment strategy, as the absence of anti-aquaporin 4 antibodies may indicate a potentially more favorable outcome and the need for a less aggressive long-term immunosuppressive therapy.

Diagnostic evaluation primarily involves an MRI of the spinal cord, which is considered the gold standard for diagnosing TM [5]. MRI can identify spinal cord lesions and inflammation, assess the length and location of the affected spinal cord segment, and rule out other structural abnormalities. CSF examination is also valuable for identifying ongoing inflammation. In our patient, both MRI and CSF findings were indicative of TM, confirming the diagnosis.

Management of TM typically begins with high-dose intravenous corticosteroids, such as methylprednisolone, to reduce inflammation and stabilize the condition [6]. Supportive rehabilitative therapies, including physical and occupational therapy, are crucial for symptom management and functional recovery. Our patient was treated with intravenous corticosteroids, which led to a noticeable alleviation of symptoms. The prognosis for older children responding to this first-line treatment is generally favorable [6]. Consistent with this, our patient showed a good prognosis following the treatment regimen.

## Conclusions

We reported the case of an 11-year-old male with a rare manifestation of TM, characterized by bilateral lower extremity weakness, sensory loss, and bowel/bladder dysfunction. MRI and CSF findings confirmed the diagnosis, ruling out other demyelinating disorders. Management with high-dose intravenous corticosteroids and rehabilitative therapies led to symptom stabilization, although some permanent deficits are anticipated due to the extent of spinal cord involvement. This case report highlights the need for prompt diagnosis and intervention in pediatric patients presenting with acute neurologic symptoms localized to the spinal cord. It adds to the existing literature by shedding further light on the classic clinical and diagnostic features of pediatric TM, thereby aiding in early recognition and management to minimize permanent disability. Future research should focus on understanding the underlying pathophysiology of this condition with an aim to develop targeted therapies.
